# Spatial access inequities and childhood immunisation uptake in Kenya

**DOI:** 10.1186/s12889-020-09486-8

**Published:** 2020-09-15

**Authors:** Noel K. Joseph, Peter M. Macharia, Paul O. Ouma, Jeremiah Mumo, Rose Jalang’o, Peter W. Wagacha, Victor O. Achieng, Eunice Ndung’u, Peter Okoth, Maria Muñiz, Yaniss Guigoz, Rocco Panciera, Nicolas Ray, Emelda A. Okiro

**Affiliations:** 1 Population Health Unit, Kenya Medical Research Institute-Wellcome Trust Research Programme, Nairobi, Kenya; 2 Health Information System Unit, Ministry of Health, Nairobi, Kenya; 3 National Vaccines and Immunization Programme, Ministry of Health, Nairobi, Kenya; 4 School of Computing and Informatics, University of Nairobi, Nairobi, Kenya; 5 Kenya Country Office, The United Nations Children’s Fund, Nairobi, Kenya; 6 Regional Office for Eastern and Southern Africa, The United Nations Children’s Fund, Nairobi, Kenya; 7 GeoHealth group, Institute of Global Health & Institute for Environmental Sciences, University of Geneva, Geneva, Switzerland; 8 Health section, The United Nations Children’s Fund, New York, USA; 9 Centre for Tropical Medicine and Global Health, Nuffield Department of Clinical Medicine, University of Oxford, Oxford, OX3 7LJ UK

**Keywords:** Spatial accessibility, Health facilities, Immunisation, Equity, Kenya

## Abstract

**Background:**

Poor access to immunisation services remains a major barrier to achieving equity and expanding vaccination coverage in many sub-Saharan African countries. In Kenya, the extent to which spatial access affects immunisation coverage is not well understood. The aim of this study was to quantify spatial accessibility to immunising health facilities and determine its influence on immunisation uptake in Kenya while controlling for potential confounders.

**Methods:**

Spatial databases of immunising facilities, road network, land use and elevation were used within a cost friction algorithim to estimate the travel time to immunising health facilities. Two travel scenarios were evaluated; (1) Walking only and (2) Optimistic scenario combining walking and motorized transport. Mean travel time to health facilities and proportions of the total population living within 1-h to the nearest immunising health facility were computed. Data from a nationally representative cross-sectional survey (KDHS 2014), was used to estimate the effect of mean travel time at survey cluster units for both fully immunised status and third dose of diphtheria-tetanus-pertussis (DPT3) vaccine using multi-level logistic regression models.

**Results:**

Nationally, the mean travel time to immunising health facilities was 63 and 40 min using the walking and the optimistic travel scenarios respectively. Seventy five percent of the total population were within one-hour of walking to an immunising health facility while 93% were within one-hour considering the optimistic scenario. There were substantial variations across the country with 62%(29/47) and 34%(16/47) of the counties with < 90% of the population within one-hour from an immunising health facility using scenarios 1 and 2 respectively. Travel times > 1-h were significantly associated with low immunisation coverage in the univariate analysis for both fully immunised status and DPT3 vaccine. Children living more than 2-h were significantly less likely to be fully immunised [AOR:0.56(0.33–0.94) and receive DPT3 [AOR:0.51(0.21–0.92) after controlling for household wealth, mother’s highest education level, parity and urban/rural residence.

**Conclusion:**

Travel time to immunising health facilities is a barrier to uptake of childhood vaccines in regions with suboptimal accessibility (> 2-h). Strategies that address access barriers in the hardest to reach communities are needed to enhance equitable access to immunisation services in Kenya.

## Background

Expanding equitable access to immunisation services and high immunisation coverage is fundamental to the progressive realization of the Sustainable Development Goal 3.8 target of universal health coverage (UHC) [1, 2]. While immunisation is one of the most cost-effective public health intervention, averting approximately 2–3 million child deaths annually, global progress has stalled in the past decade [3, 4]. By 2018, only 129 (66%) countries had achieved 90% national coverage of three doses of diphtheria-tetanus-pertussis (DTP3) vaccine leaving about 19.4 million infants vulnerable to vaccine-preventable diseases (VPDs), majority (44%) of whom were in sub- Saharan Africa (SSA) [5].

SSA has an estimated 8.5 million un/under-vaccinated children [5]. The region faces specific challenges including rapid population growth, increasing urban poor communities and slums and weak health systems making it even more challenging to sustain current levels of coverage [6, 7]. Multiple indicators are critical to the comprehensive assessment of the extent of inequities and to inform targeted approaches [8]. Across all country signatories to Global Vaccine Action Plan, geographic disparity in immunisation coverage has been a main indicator for tracking and measuring equity with the goal of reaching 80% coverage of DPT3 in every district or equivalent sub-national administrative unit [3, 9].

Many barriers including supply-side factors such as availability of commodities and accessibility to health service and demand-side factors at the contextual and individual level, contribute to unequal vaccination coverage within a country [10]. Studies in SSA have shown that factors such as maternal education, exposure to media, family size, household wealth, antenatal care service utilization, access to health facilities significantly affect immunisation coverage [11–16].

Multiple contacts with health facilities are required to achieve full immunisation status [17]. However, long distances and increased travel times (spatial accessibility) remains a major barrier to expanding immunisation coverage in many SSA countries [15, 18, 19]. This is more pronounced in the rural areas, where most of the unvaccinated children live and where services are limited [20, 21] and in informal settlement that are densely populated and often affected by significant traffic delays [22, 23]. Although the influence of different factors on access to health care and immunisation services has previously been assessed, the local context determines how these factors interact. Hence, from a programme perspective, it is important to understand locally how the interplay between the various factors impacts vaccination coverage.

Kenya has made remarkable progress in improving accessibility and provision of immunisation services since the adoption of Kenya Expanded Programme on Immunisation (KEPI) in 1980 [24]. However, there is substantial geographic heterogeneity in the coverage of full immunisation ranging from 31.2–92.3% across counties, the current health planning units in Kenya [25]. To achieve the UHC target of ‘vaccines for all with at least 90% coverage’, the Kenya Health Sector Strategic and Investment Plan (KHSSIP) policy highlights the importance of ensuring that essential health services are available with a focus on improving spatial access [26]. The target is to ensure that health service provision sites are within 5 km (one-hour) of walking distance of every individual residence [27, 28] and that 90% of the total population is within 5 km of public health services [26]. Yet, the role of spatial access to primary health services is poorly described in Kenya with only a few localized studies evaluating the effect of spatial access on child immunisation [29, 30].

In this study, we estimate the travel time to health facilities offering immunisation services in Kenya using a spatial database of geocoded health facilities, road network, digital elevation model and land use within a geospatial framework and assess its effect on immunisation coverage measured at survey clusters using multi-level logistic regression.

## Methods

### Defining spatial access to immunisation health facilities

#### Health facilities

Health facilities that offer immunisation services were sourced from the Kenya Master Health Facility List (KMHFL) [31] and the Kenya Health Information System (KHIS) [31, 32] based on the District Health Information Systems version 2(DHIS2). Facilities that offer immunisation services based on the reported number of vaccinations during 2012–2014 period were identified from the KHIS and merged to KMHFL list to obtain coordinates. Where coordinates were missing, a previously geocoded health facility list was used [33]. The final list of health facilities obtained covered the whole spectrum of health facility levels and ownership status, comprising both public and private facilities.

#### Ancillary datasets

Relevant ancillary datasets of factors that influence travel speeds including road network, land cover and digital elevation model (DEM) were assembled nationally. Road network data was obtained from the ministry of transport of Kenya that used the gold standard GPS technique to map coverage of roads in 2016 [34]. This was overlaid with roads obtained from OpenStreetMaps (OSM) and Google Map Maker (GMM) [35, 36] and combined using ArcMap version 10.5 (ESRI Inc., Redlands, CA, USA). We eliminated duplicates, corrected for road sections with short connection segments due to digitization and deleted those that extended to water bodies from the resultant vector file. Roads were classified as primary, secondary, county and rural roads [37]. Land cover was based on 2016 Copernicus Sentinel-2 satellites at 20 m × 20 m spatial resolution available from RCMRD GeoPortal [38]. It contained five land cover categories namely; bare areas, built up areas, water bodies, cultivated areas and vegetation cover areas (forests, shrubs and grassland areas). Major rivers and lakes available from global lakes and wetlands database [39] were considered as barriers to movement (except in the presence of bridges informed by the road data set). Forty-nine protected areas [40–42] were considered unpassable and treated as barriers as shown in the additional file 1. The DEM from Shuttle Radar Topographic Mission (SRTM) 30 m × 30 m spatial resolution archived at RCMRD Geoportal [38] was used to account for the influence of topography on walking and bicycling speeds.

#### Population data

Several modelled population distribution datasets exist [43]. However, these datasets include covariates such as the density of health facilities and road networks to determine regions likely to be inhabited. To avoid model induced correlation, such as circularity when estimating travel time to health facilities, a population distribution map that excluded density of health facilities and road networks was constructed using dasymetric spatial modelling techniques. Kenya’s 2009 census population data were redistributed at enumeration areas (EA) to 100 m square grids and projected to 2014. During the redistribution, the grids were weighted based on the probability of being inhabited and the relationship between population density [44]. The weights were then used in a random forest technique while adjusting for rural-urban differences to obtain population at the 100 m square grids [45].

#### Computing travel time

Landuse, road network and travel barriers (protected areas and water bodies) were rasterized, resampled to 100 m square grids and combined in ArcMap 10.5 (ESRI Inc., Redlands, CA, USA). The resultant raster was used to generate a cost raster surface with impedance value based on cumulative speeds at each 100 m square grids predetermined spatial grids. Travel time to the nearest health facility was computed using the generated cost surface and locations of the health facilities through each square grids from all areas in Kenya on a regular raster grid using AccessMod 5.0 [46, 47]. Slope derived from DEM was used to adjust walking speeds using Tobler’s formulation [48] and to adjust for bicycling speeds using bicycling power correction [49, 50].

Two possible travel scenarios typically used by the Kenyan population to access health facilities were assessed; one where we assume walking only scenario and a second more optimistic travel scenario that assumed the population walks to the nearest road and takes a different mode of transport immediately available depending on the terrain and the available road infrastructure as shown in additional file 2. The walking scenario was important since most of the population use walking as the main mode of transport, especially in the rural areas [28, 51] where about 73% of people reside in Kenya [52]. In addition, it facilitated the evaluation of the government policy, for a threshold of 90% of the people within an hour of walking to the nearest facility [26]. Input travel speeds for each road type and landcover were adopted from previous work in Kenya [37, 51] and refined through a discussion with the National Vaccination and Immunization Programme (NVIP) staff from five counties and the national offices in Kenya [53].

The output of the accessibility analysis was two continuous surfaces depicting the theoretical time it would take to get to the nearest immunising health facility for walking only and a combination of walking and motorized travel models. The travel time was depicted in minutes at a spatial resolution of 100 m square grids for the entire country.

The geographical coordinates for sampled clusters in Kenya Demographic Health Survey 2014 (KDHS 2014) were used to extract travel times for each child needing immunisation. Since the cluster coordinates are randomly perturbed by up to 5 km in rural areas and 2 km in urban areas [54], 5 km and 2 km buffers were drawn around the rural and urban clusters respectively and mean travel times extracted within the buffers. Maps of travel time to the nearest immunising health facility at 100 m square grids and the average time per cluster were then plotted in ArcMap version 10.5 (ESRI Inc., Redlands, CA, USA). Using the continuous travel time surfaces from the walking only and the optimistic cost analysis, we computed the proportion of the total population at county levelwithin 1-h to the nearest immunising health facility.

### Outcome variables

Data on immunisation coverage for children aged 12–23 months and its predictors collected from women aged 15–49 were based on KDHS 2014 conducted between May and October 2014. KDHS 2014 employed a two-stage sampling design where 1612 clusters were selected in the first stage while 40,300 households were selected in a second stage [25]. This is the largest sample household survey to ever be conducted in Kenya.

The main outcomes were DPT3 vaccination status and fully immunised child (FIC) status defined by KEPI [55] as having received: one dose each for Bacille Calmette-Guerin (BCG) and measles, DPT3, polio (excluding polio at birth) and pneumococcal vaccines from either the child’s vaccination card or by mother’s recall for children aged 12–23 months. Fully immunised status was used to assess the overall impact of travel time to health facilities on immunization coverage. The antigens contained in FIC status are either delivered through stationary health facilities or via supplemental immuinisation activities. Therefore, the effect of travel time might be diminished. To mitigate against the attenuated effect, DPT3 vaccine was used to validate the impact of travel time to health facilities because it is offered through stationary health facilities which are not influenced by supplemental immunisation activities.

### Confounding variables

Computed travel time to the nearest immunising health facility was the primary explanatory variable of interest. Potential confounders found in the literature related both utilization of healthcare services and travel time to health facilities were identified, reviewed and included based on data availability. Data were abstracted from KDHS 2014. The covariates considered as confounders were mother’s education level, person who decides on mothers/child healthcare seeking, parity, residence type, marital status, mother’s age and household wealth index [56] . Each variable was categorized based on a literature review assessing the association between immunisation coverage and its determinants [11–16]. Among the abstracted confounders, wealth index describing social economic status across households and parity were derived from a combination of several indicators. Wealth quintiles (index) derived from the DHS measures the relative socioeconomic status of households based on household assets and amenities at the time of the survey using principal component analysis. The wealth quintiles were classified into poorest, poor, middle, rich and richest [57]. Since maternal age and parity are highly correlated, parity was categorized into two groups as follows; high parity (if mother’s age < 30 years and has more than two children living in the household or is aged ≥30 years and has more than three children living in the household and low parity otherwise. Although datasets may be available from other sources, we restricted the sample to KDHS 2014 for consistency and a common period.

### Statistical analysis

Proportions were computed to describe the characteristics of data in relation to FIC status and DPT3. We estimated crude associations between the dependent or confounding factors and the two outcomes FIC status and DPT3. Confounders that were significant at the cutoff (*p* < 0·20) in the crude analysis were incorporated into the multivariable regression analysis. The Person who decides on mothers/child healthcare seeking variable was excluded from the analysis as it was asked for a subset of women (married mothers) in the sample. Computed travel time from the optimistic scenario was used in the analysis as it has been shown to provide a more realistic estimate of travel times [58]. We anticipated unmeasured effects at the county, community and individual levels due to the hierarchical data structure of DHS data [57]. A null model with no explanatory variables was fit and the intracommunity correlation coefficient (ICC) was used to assess clustering at county and cluster levels. Therefore, we conducted the analysis with counties as level 1, clusters as level 2 and individuals (children) as level 3.

A multi-level logistic regression model was used to assess the relationship between immunisation coverage and travel time to immuinisation facility when controlling for potential confounders. Travel time to health immunising facilities was added first followed by the confounders in an increasing order of their *p* values from bivariate analysis. We also examined statistical interactions based on a priori hypotheses that the effect of travel time to immunising health facility may be different for rich and poor households as well as between travel time and urban/rural residence.

To define the final model, we reported adjusted odds ratios with their 95% confidence intervals, and Wald test (*p*-value < 0.05) to inform the overall significance of the models. Multicollinearity test to evaluate associations among the independent variables was assessed using Variance Inflation Factor (VIF) at cut off point of 5 [59]. The analyses were done using STATA v.14 (Stata Statistical Software: Release 14. College Station, TX: StataCorp LP) and KDHS 2014 sampling weights were incorporated throughout the analysis.

## Results

### Travel time to health facilities

The current travel time analysis involved 6135 (98%) of the identified vaccination sites. Despite several attempts at geocoding, we could not geo-locate 96 health facilities. In addition, 18 health facilities within protected areas considered as barriers to travelling were excluded from the analysis. Of the 6135 health facilities, 72% (4436) were public while 28% (1699) were private health facilities. Majority of the facilities, 72% (4445), were level 2 (primary health care facilities).

**Fig. 1:**
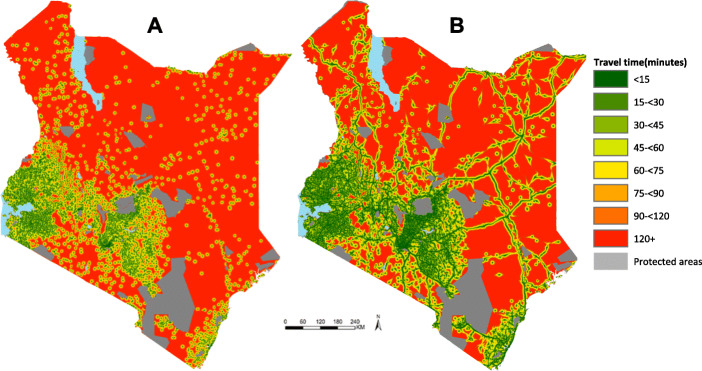
Spatial accessibility to the nearest immunising health facilities in Kenya based on two travelling scenarios. **a** Scenario 1; Walking only **b** Scenario 2; Optmistic (a combination of walking and any motorized transport). Travel time is categorized into 15 min bands ranging from < 15 min (dark green) to 120+ minutes (red). Grey areas represent protected areas whose names and designate are provided in additional file 1. (Source: Authors)

Figure 1 provides a visual representation of geographic access to the nearest immunising health facility in Kenya using two travel scenarios; scenario 1 (Map A) (walking only) and scenario 2 (Map B) (an optimistic scenario that combined walking and motorized transport).

### Walking scenario only

The national mean travel time to immunising health facilities was 63 min ranging from 11 min in Nairobi county to 171 min in Marsabit county. Only 75% of the total population in Kenya lived within < 1-h to the nearest immunising health facility falling short of the government’s 90% target. The national value masked a lot of heterogeneity at the county level. The proportion of the total population with access to immunising health facilities ranged from 21% in Samburu county to 100% in Nairobi, Vihiga, Kisii and Nyamira counties. Eighteen counties (38%) had achieved the target of > 90% of the total population living < 1-h. The most marginalized counties were Wajir, Mandera Turkana and Samburu counties with < 30% of the population living < 1-h of the nearest immunising health facility.

### Walking and motorized scenario

**Fig. 2:**
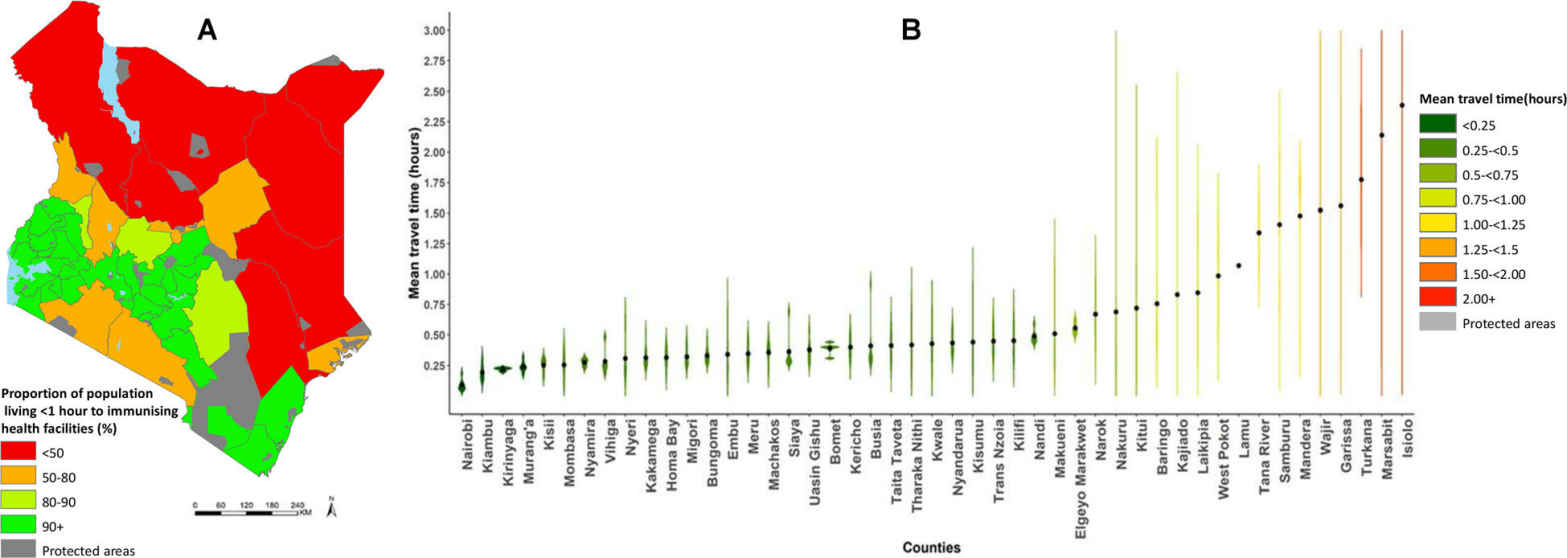
Catchment population and travel time at county units. **a** Proportion (%) of catchment population within one-hour to immunising health facilities at county units based on the optimistic travel scenario. Proportion estimates are grouped into four categories as follows; < 50% (red), 50- < 80(brown), 80- < 90 (light green) and 90+ (green) and grey areas represent protected areas whose names and designate are provided in additional file 1**b** Distribution of travel times within each county capped at 2 h using the optimistic travel scenario. Height of the bars indicate the level of variation in travel time within a county. Scatter dots represent mean travel time in each county. (Source: Authors)

In contrast, using the optimistic scenario (a combination of walking and motorized transport) the national mean travel time to an immunisation facility was 40 min. Overall, 93% of the total population lived within < 1-h travel time to the nearest immunizing health facility with substantial variations across counties ranging from 38% in Turkana county to 100% in Nairobi, Vihiga, Kisii, Nyamira, Kirinyaga, Mombasa, Migori, Murang’a, Busia, Kisumu, Bomet, Kiambu, Bungoma, Siaya, Homabay and Kericho counties. Thirty four percent (16) counties had less than 90% of the total population living < 1-h to immunising health facility (Fig. 2a). Figure 2b shows the distribution of mean travel time across counties using the combined walking and motorized transport. Nairobi county and Isiolo county had the highest and the lowest mean travel time of 5 min and 2-h respectively. Seven counties in Eastern (Isiolo and Marsabit), North Eastern (Garissa, Wajir and Mandera), Rift valley (Samburu and Turkana and Coast (Tana River) regions were most marginalized with travel times ranging from 84 min to 2 h.

### Descriptive analysis

The immunisation status of 75% (3047) of the children was obtained from child vaccination cards, and that of 25% (1005) of the children was determined from the mother’s recall. Of the 4052 children aged 12–23 months included in the analysis, 76% [95% CI: 75–77%] were fully immunized, and 90% [89–91%] had received DPT3 vaccine at the time of the survey. The summary statistics and bivariate associations are presented in Table 1. The overall mean travel time for KDHS 2014 clusters was 35 min (95% CI: 33–37). Eighty-seven per cent (1229 of 1406) of the clusters had mean travel time < 1-h to the nearest immunising health facilities (additional file 3).

**Table 1:** Descriptive and univariate analysis results (unadjusted odds ratios) for travel time to health facilities and confounding determinants associated with full immunisation status and DPT3 vaccine for children aged 12–23 months during the 2014 Kenya Demographic and Health Survey

Determinants	Fully immunised n(%)	Not fully immunised n(%)	Unadjusted OR (95% CI)	*p* value	Received DPT3 n(%)	Didn’t receive DPT3 n(%)	Unadjusted OR (95% CI)	*p* value
**Mean travel time (minutes)**								
< 30	2018 (79.3)	521 (20.7)	1		2335 (91.5)	199 (8.5)	1	
30- < 60	581 (70.2)	242 (29.8)	0.76 (0.56–1.02)	0.071	737 (88.9)	88 (11.1)	0.93 (0.63–1.38)	0.728
60- < 90	161 (58.6)	102 (41.5)	0.47 (0.32–0.68)	< 0.0001	228 (85.7)	38 (14.4)	0.67 (0.39–1.17)	0.16
90- < 120	85 (60.8)	81 (39.2)	0.44 (0.20–0.96)	0.039	121 (79.9)	44 (20.1)	0.26 (0.12–0.56)	0.001
120+	127 (53.8)	11 (46.2)	0.34 (0.21–0.54)	< 0.0001	175 (73.3)	64 (26.7)	0.22 (0.12–0.42)	< 0.0001
**Maternal education**								
No education	488 (56.5)	321 (43.5)	1		677 (77.7)	188 (22.3)	1	
less than secondary	1880 (75.6)	416 (24.4)	2.30 (1.59–3.32)	< 0.0001	2270 (91.1)	217 (8.9)	3.97 (2.16–7.30)	< 0.0001
secondary or higher	618 (86.2)	42 (13.8)	4.65 (3.17–6.80)	< 0.0001	668 (93.3)	32 (6.7)	5.73 (3.32–9.89)	< 0.0001
**Household wealth index**								
Poor	885 (63.0)	497 (37.0)	1		1189 (83.7)	247 (16.4)	1	
Least poor	655 (76.1)	153 (23.9)	1.61 (1.07–2.43)	0.022	778 (90.4)	75 (9.6)	2.05 (1.18–3.58)	0.015
Middle	523 (79.1)	87 (20.9)	2.1 (1.63–2.69)	< 0.0001	605 (92.0)	42 (8.0)	1.69 (1.11–2.60)	0.011
Rich	429 (83.0)	37 (17.0)	3.01 (1.91–4.74)	< 0.0001	558 (93.9)	40 (6.1)	4.17 (1.18–3.58)	< 0.0001
Richest	431 (82.6)	5 (17.4)	3.3 (2.28–4.79)	< 0.0001	485 (92.8)	33 (7.2)	4.48 (2.48–8.10)	< 0.0001
**Parity**								
Low	1726 (81.9)	429 (18.1)	1		1999 (93.2)	156 (6.8)	1	
High^a^	1260 (67.3)	637 (32.7)	0.50 (0.38–0.64)	< 0.0001	1616 (85.7)	281 (14.3)	0.41 (0.29–0.59)	< 0.0001
**Type of residence**								
Urban	974 (78.8)	287 (21.2)	1		1150 (91.3)	11 (8.6)	1	
Rural	2012 (74.3)	779 (25.7)	0.80 (0.63–1.03)	0.08	2465 (89.4)	326 (10.6)	0.73 (0.51–1.05)	0.089
**Mother’s age (years)**								
≤ 20	359 (77.3)	84 (22.7)	1		436 (93.5)	44 (6.6)	1	
21–30	1770 (76.7)	439 (23.3)	0.93 (0.63–1.38)	0.728	2145 (90.5)	245 (9.5)	0.57 (0.31–1.07)	0.081
≥ 31	857 (73.5)	256 (26.5)	0.77 (0.50–1.17)	0.22	1034 (87.8)	148 (12.2)	0.37 (0.19–0.74)	0.005
**Mother’s marital status**								
Married/living with a partner	2533 (75.5)	679 (24.5)	1		3074 (90.0)	374 (10.0)	1	
Widowed/divorced/separated	225 (76.5)	57 (23.5)	0.94 (0.55–1.61)	0.832	269 (87.5)	46 (12.5)	0.66 (0.28–1.55)	0.336
Never in union	228 (79.5)	43 (20.5)	1.04 (0.69–1.57)	0.854	272 (93.8)	17 (6.2)	1.57 (0.78–3.14)	0.204

^a^ High parity (if mother’s age < 30 years and has more than two children living in the household or is aged ≥30 years and has more than three children living in the household and low parity otherwise

Children living in regions with a mean travel time < 1-h had significantly higher immunisation coverage for both DPT3 (91%) and fully immunised status (78%) compared to those who lived > 1 h from the immunising health facility with coverage of 82 and 60% respectively (*p* value< 0.001). Additionally, 2 % of the children living within less than one-hour did not receive any dose of DPT3 vaccines compared to 6 % who lived more than one-hour from the immunising health facility (p value = 0.08).

### Travel time and childhood immunisation status

#### Bivariate analysis

Increased mean travel time to a health facility was associated with reduced odds of being fully immunised; 30- < 60 min (crude OR = 0.76, *p* = 0.071), 60- < 90 min (crude OR = 0.47, *p* < 0.0001), 90- < 120 min (crude OR = 0.44, *p* = 0.039), 120+ minutes (crude OR = 0.34, *p* < 0.0001). A similar trend was observed for DPT3 coverage with increased mean travel time associated with relatively smaller likelihood of a child getting vaccinated compared to that of fully immunised outcome; 30- < 60 min (crude OR = 0.93, *p* = 0.728), 60- < 90 min (crude OR = 0.67, *p* = 0.16), 90- < 120 min (crude OR = 0.26, *p* = 0.001), 120+ minutes (crude OR = 0.22, p < 0.0001).

#### Multivariable analysis

The null model revealed significant variability of fully immunised status (τ = 0.76, *p* < 0.001) and DPT3 (τ = 1.58, *p* < 0.001) across counties and clusters. The ICC showed that 31 and 43% of the variability in odds of fully immunised status and DPT3 was due to county and cluster level differences. After adjusting for confounding variables, the variation in the odd of fully immunised status (τ = 0.84, *p* < 0.001) and DPT3 (τ = 1.68, *p* < 0.001) remained significant. At the same time, 25 and 39% of the variance in fully immunised status and DPT3 among children was due to county and cluster level factors.

Mean travel time of up to 120+ minutes was significantly associated with fully immunised status (AOR = 0.56[0.33–0.94]) and DPT3 coverage [AOR = 0.51(0.21–0.92)] after adjusting for mother’s education level, household wealth, parity and urban/rural residence. However, this effect was not significant for short to moderate travel time (minutes); 30- < 60;[AOR:0.95(0.67–1.35)], 60- < 90;[AOR:0.77(0.52–1.19)] and 90- < 120; [AOR: 0.67 (0.30–2.40)] for fully immunised status and 30- < 60;[AOR:1.44(0.91–2.29)], 60- < 90;[AOR:1.45(0.79–2.69)] and 90- < 120; [AOR: 0.54 (0.24–1.12)] for DPT3 vaccine (Table 2). We did not adjust for marital status and mother’s age for fully immunised outcome in the multivariable model as they were not significant at *p* < 0.20 in the univariate model. On the other hand, mother’s age was significant for DPT3 vaccine but collinear with parity and was excluded (VIF = 11.2). No significant interactions effects between travel time and household wealth as well as between travel time and urban/rural residence were found.

**Table 2:** Multivariate multi-level logistic regression model adjusted odds ratios of mean travel time tohealth facilities while controlling for confounding determinants associated to full immunisation and DPT3 vaccine among children aged 12–23 months during the 2014 Kenya Demographic and Health Survey

Determinants	Fully immunised		Received DPT3 vaccine	
	n(%)	AOR^a^ (95% CI^b^)	n(%)	AOR (95% CI)
**Mean travel time (minutes)**				
< 30	2018 (79.3)	1	2335 (91.5)	1
30- < 60	581 (70.2)	0.95 (0.67–1.35)	737 (88.9)	1.44 (0.91–2.29)
60- < 90	161 (58.6)	0.77 (0.52–1.19)	228 (85,7)	1.45 (0.79–2.69)
90- < 120	85 (60.8)	0.67 (0.30–2.40)	121 (79.9)	0.54 (0.24–1.21)
120+	127 (53.8)	0.56 (0.33–0.94) **	175 (73.3)	0.51 (0.21–0.92) **
**Maternal education**				
No education	488 (56.5)	1	677 (77.7)	1
less than secondary	1880 (75.6)	1.55 (1.00–2.40) **	2270 (91.1)	2.59 (1.25–5.38) **
secondary or higher	618 (86.2)	2.34 (1.46–3.75) ***	668 (93.3)	2.44 (1.14–5.22) **
**Household wealth index**				
Poor	885 (63.0)	1	1189 (83.7)	1
Least poor	655 (76.1)	1.36 (0.89–2.10)	778 (90.4)	1.30 (0.83–2.02)
Middle	523 (79.1)	1.67 (1.24–2.25) ***	605 (92.0)	1.57 (0.89–2.78)
Rich	429 (83.0)	2.23 (1.32–3.76) ***	558 (93.9)	3.24 (1.58–6.63) ***
Richest	431 (82.6)	2.32 (1.43–3.76) ***	485 (92.8)	3.48 (1.72–7.08) ***
**Parity** ^c^				
Low	1726 (81.9)	1	1999 (93.2)	1
High	1260 (67.3)	0.60 (0.46–0.78) ***	1616 (85.7)	0.51 (0.35–0.74) ***
**Type of residence**				
Urban	974 (78.8)	1	1150 (91.3)	1
Rural	2012 (74.3)	1.39 (1.06–1.82) **	2465 (89.4)	1.47 (1.02–2.28) **
***Random effects***				
County & cluster variance (SE) ^d^		0.84 (0.34)		1.68 (0.67)
ICC ^e^		0.25		0.39
Wald test		< 0.0001		< 0.0001
Mean VIF ^f^		1.2		1.3

*P < 0.1, **P < 0.05, ***P < 0.01, Adjusted odds ratios, ^b^ Confidence interval, ^c^ High parity (if mother’s age < 30 years and has more than two children living in the household or is aged ≥30 years and has more than three children living in the household and low parity otherwise ^d^ Standard error, ^e^ Intra-class correlation coefficient, ^g^ Variance inflation factor

## Discussion

There has been considerable progress in increasing immunisation coverage, yet equity remains an area of focus. We evaluated the contribution of travel time equity gaps to immunisation status for prioritization. There was substantial variation in spatial access across the country. Sixty two percent (29 of 47) of the counties did not meet the current Kenya policy target of 90% of the population living within 1 h (walking speeds) of a health facility offering immunisation services [26]. Assuming an optimistic travel time scenario, 66% (31 of 47) of the counties had at least 90% of the population living within 1 h from a health facility. Using this scenario, counties in North Eastern (Wajir, Mandera and Garissa) parts of Eastern and Coast (Marsabit, Tanariver) and Rift Valley (Samburu and Turkana) regions were identified as the most marginalized regions with less than 50% of the total population living less than one-hour to a health facility. There was a co-location between counties with poor access and low immunisation coverage. For example, counties with full immunisation coverage less than 50% (Wajir and Mandera) [57] had mean travel times of more than an hour to immunising health facilities while 6 % of children 12–23 months living in clusters with mean travel time more than one-hour did not receive any dose of DPT vaccine.

The mean travel time to health facilities offering immunisation services was 40 min, with 7 % of the total population in Kenya living more than one-hour away from an immunising health facility assuming that populations use a combination of walking and motorized/cycling transport. Previous studies [58, 60, 61] show that using the optimistic travel scenario provides a robust analytical spatial technique to estimating travel times. However, estimates from the walking scenario are useful for highlighting limitations due to inadequacies of the road infrastructure and availability of public transport as well as evaluating travel time targets in Kenya [28, 62]. Assuming that care givers of children needing vaccinations walked to a health facility, the mean travel time to the nearest health facilities was 63 min, an increase of nearly half an hour travel time compared to the optmistic travel scenario. This translates to 25% of the population living more than 1 h away for an immunising health facility. Wajir, Mandera, Turkana and Samburu had significant proportions of their populated areas outside the one-hour band with 58% (walking and motorised scenario) and 77% (walking scenario) of the population living more than one-hour from a facility offering immunisation services respectively. The maps demonstrate the significant role played by adequate road networks in expanding accessibility, with access being high in areas covered by extensive road network.

Univariate analysis indicated a pronounced decline in coverage of immunisation with increased travel time to health facilities for travel times of greater than an hour. Longer travel time also correlate with other factors such as increased transport costs which may also play a major role in decreased service utilization [15, 19]. There was a significant effect on both fully immunised status and DPT3 coverage up to more than two-hours. Observed lower odds of longer travel time on DPT3 compared to fully immunised status highlight a differential access problem since infants require three contacts with the health facility to receive DPT3 vaccine, which is typically delivered through stationary health posts unlike fully immunised status that is influenced by supplemental immunisation activities for measles and polio vaccines [63]. Across SSA, the effect of spatial access on immunisation uptake has been varied. Some studies found travel time to health facilities to be an important determinant of child immunisation [15, 18, 19, 30, 64] while others did not find a significant association [29, 65, 66]. These varied findings were attributed to intra-regional disparities between urban and rural regions depicting an ‘urban advantage’ due to high density of health centers that reduced the effect of travel time on child immunisation outcomes. However, urban advantage in access to healthcare services including childhood immunisation is significantly minimized or reversed after adjusting for wealth since poverty; a previously predominant phenomenon in rural areas, is increasing in urban settings especially in urban slums [67]. Complementary strategies such as promotional health education campaigns through customized media programmes, addressing opportunity costs especially for the poor such as, initiating conditional cash transfer programs has been successfully demonstrated in other countries [68] in addition to ensuring proximity to health facilities would enhance access to immunisation services [19, 69].

There were several limitations to our study. First, spatial access metric was computed using the nearest immunising health facility since KDHS do not report the actual health facilities used to access immunisation services. However, it is possible that a given proportion of the population does not use the nearest health facilities due to perceived quality of services [70] or effects of health system functions such as stock-outs and strikes [71]. Secondly, the two gridded surfaces of travel time did not account for seasonality [60, 72] nor traffic delays [22, 73] limited by the unavailability of data on road conditions and traffic congestion given the geographic scope of the analysis. Time needed to get to the nearest health facility could vary greatly during rainy seasons due to flooding making roads impassable or decreasing travelling speeds [72, 74] while traffic flow variability influence travel time, especially in urban regions [22, 23]. Third, there may have been misclassification of travel time computed based on 2016 ancillary datasets that were assigned to KDHS 2014. The clusters coordinates are randomly perturbed for confidentiality, consequently 5 km and 2 km buffers were drawn around the rural and urban clusters to minimize the effects of scrambling of coordinates, however this does not fully account for the scrambled coordinates. Fourth, respondent-based reported factors were used to identify determinants of child immunisation; therefore, models in this analysis do not account for provider-related factors associated with immunisation uptake such as waiting times, quality of services, stock-outs and strikes. Finally, the inclusion of data obtained from the mother’s recall of immunisation information may have introduced recall bias. However, it has been shown that mother’s vaccination recall can be a good measure of child immunisation indicators, especially for the youngest child cohort (12–23 months) minimising the effect of recall bias [25, 57]**.**

## Conclusion

In conclusion, substantial inequities in spatial access persist within the country. This analysis provides a basis for better-informed resource allocation at units below the county level that can serve to mitigate inequalities in spatial accessibility and reach marginalized populations. County governments and implementing agencies can use such spatial access outputs to enhance the achievement of GAVI’s strategy post-2019 period [75] that focuses on addressing equity. Strategies focused on strengthening routine immunisation services such as mobile clinics to counties with considerable low accessibility while addressing opportunity costs especially for the poor are crucial to improving child immunisation outcomes in Kenya.

## Supplementary information

**Additional file 1:.** A list of protected areas and their designate reservation purpose considered impassable by the population target during modelling of travel time to immunising health facilities.

**Additional file 2:.** Description of land cover types, mode of travel (walking, vehicular and bicycling) and speeds used in modelling travel time to immunising health facilities.

**Additional file 3:.** A map showing mean travel time to the nearest heath facility using the combined walking and motorized travel scenario categorized into 15 min bands ranging from < 15 min (dark green) to 120+ minutes (red) for the sampled KDHS 2014 clusters.

## Data Availability

The KDHS dataset is publicly available to registered users on the Demographic and Health Surveys website while all the other datasets are publicily available through the web links provided in the manuscript.

## Abbreviations

KEPI: Kenya Expanded Programme on Immunisation
FIC: Fully immunised child
DPT3: The third dose of diphtheria-tetanus-pertussis vaccine
KHIS: Kenya Health Information System
KMHFL: Kenya Master Health Facility List
DEM: Digital Elevation Model
RCMRD: Regional Centre for Mapping of Resources for Development
