# Supplementary material for: Spatial access inequities and childhood immunisation uptake in Kenya
Source: BMC Public Health. 2020 Sep 15;20:1407. doi: 10.1186/s12889-020-09486-8 (PMC7493983; doi:10.1186/s12889-020-09486-8)
Supplement: Supplementary file 1 — Additional file 1:. A list of protected areas and their designate reservation purpose considered impassable by the population target during modelling of travel time to immunising health facilities. [file 12889_2020_9486_MOESM1_ESM.docx]

| **Name** | **Designate** |
| --- | --- |
| Masai Mara | National Reserve |
| South Island | National Park |
| Sibiloi | National Park |
| Central Island | National Park |
| Marsabit | National Reserve |
| South Turkana | National Reserve |
| Mount Elgon | National Park |
| Kerio Valley | National Reserve |
| Shaba | National Reserve |
| Samburu | National Reserve |
| Kakamega | National Reserve |
| Maralai | Game Sanctuary |
| Ruma | National Park |
| Tsavo East | National Park |
| Shimba Hills | National Reserve |
| Arawale | National Reserve |
| Chyulu | National Park |
| Amboseli | National Park |
| Losai | National Reserve |
| Nasolot | National Reserve |
| Buffalo Springs | National Reserve |
| Bisanadi | National Reserve |
| Meru | National Park |
| Kora | National Park |
| North Kitui | National Reserve |
| Mwea | National Reserve |
| Ol Donyo Sabuk | National Park |
| Ngai Ndethya | National Reserve |
| Boni | National Reserve |
| South Kitui | National Reserve |
| Tsavo West | National Park |
| Tana River Primate | National Reserve |
| Nairobi | National Park |
| Dodori | National Reserve |
| Arabuko Sokoke | Forest Reserve |
| Longonot | National Park |
| Aberdare | National Park |
| Hell's Gate | National Park |
| Lake Bogoria | National Reserve |
| Lake Nakuru | National Park |
| Mombasa | Marine National Reserve |
| Watamu | Marine National Park |
| Mpunguti | Marine National Reserve |
| Kisite | Marine National Park |
| Kiunga | Marine National Reserve |
| Malindi | Marine National Park |
| Diani-Chale | Marine National Reserve |
| Mount Kenya | National Park |
| Malka Mari | National Park |

A list of protected areas and their designate reservation purpose considered impassable by the population target during modelling of travel time to immunising health facilities
