# Supplementary material for: Spatial access inequities and childhood immunisation uptake in Kenya
Source: BMC Public Health. 2020 Sep 15;20:1407. doi: 10.1186/s12889-020-09486-8 (PMC7493983; doi:10.1186/s12889-020-09486-8)
Supplement: Supplementary file 2 — Additional file 2:. Description of land cover types, mode of travel (walking, vehicular and bicycling) and speeds used in modelling travel time to immunising health facilities. [file 12889_2020_9486_MOESM2_ESM.docx]

| **Land cover category** | **Speed (km/h)** | **Mode of transport** |
| --- | --- | --- |
| Trees cover areas | 2 | Walking |
| Shrubs cover areas | 4 | Walking |
| Grassland | 3 | Walking |
| Cropland | 4 | Walking |
| Vegetation aquatic or regularly flooded | 2 | Walking |
| Lichen Mosses and Sparse vegetation | 4 | Walking |
| Bare areas | 4 | Walking |
| Built up areas | 4 | Walking |
| Open water | 0.01 | Walking |
| Primary roads | 50 | Vehicular |
| Secondary roads | 30 | Vehicular |
| County roads | 11 | Bicycling |
| Rural roads | 5 | Walking |

Description of land cover types, mode of travel (walking, vehicular and bicycling) and speeds used in modelling travel time to immunising health facilities.

*Vehicular mode of transport includes motorcycle
