# Supplementary material for: Spatial access inequities and childhood immunisation uptake in Kenya
Source: BMC Public Health. 2020 Sep 15;20:1407. doi: 10.1186/s12889-020-09486-8 (PMC7493983; doi:10.1186/s12889-020-09486-8)

A map showing mean travel time to the nearest heath facility using the combined walking and motorized travel scenario categorized into 15 minutes bands ranging from <15 minutes (dark green) to 120+ minutes(red) for the sampled KDHS 2014 clusters.

**Travel time (minutes)**

<15

15-<30

30-<45

45-<60

60-<75

75-<90

90-<120

120+


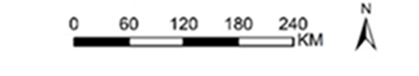

Supplement: Supplementary file 3 — Additional file 3:. A map showing mean travel time to the nearest heath facility using the combined walking and motorized travel scenario categorized into 15 min bands ranging from < 15 min (dark green) to 120+ minutes (red) for the sampled KDHS 2014 clusters. [file 12889_2020_9486_MOESM3_ESM.docx]
